# A digital mask to safeguard patient privacy

**DOI:** 10.1038/s41591-022-01966-1

**Published:** 2022-09-15

**Authors:** Yahan Yang, Junfeng Lyu, Ruixin Wang, Quan Wen, Lanqin Zhao, Wenben Chen, Shaowei Bi, Jie Meng, Keli Mao, Yu Xiao, Yingying Liang, Danqi Zeng, Zijing Du, Yuxuan Wu, Tingxin Cui, Lixue Liu, Wai Cheng Iao, Xiaoyan Li, Carol Y. Cheung, Jianhua Zhou, Youjin Hu, Lai Wei, Iat Fan Lai, Xinping Yu, Jingchang Chen, Zhonghao Wang, Zhen Mao, Huijing Ye, Wei Xiao, Huasheng Yang, Danping Huang, Xiaoming Lin, Wei-shi Zheng, Ruixuan Wang, Patrick Yu-Wai-Man, Feng Xu, Qionghai Dai, Haotian Lin

**Affiliations:** 1 State Key Laboratory of Ophthalmology, Zhongshan Ophthalmic Center, Sun Yat-sen University, Guangdong Provincial Key Laboratory of Ophthalmology and Vision Science, Guangdong Provincial Clinical Research Center for Ocular Diseases, Guangzhou, China; 2 School of Software and BNRist, Tsinghua University, Beijing, China; 3 Department of Ophthalmology, Guangdong Provincial People’s Hospital; Guangdong Academy of Medical Sciences, Southern Medical University, Guangzhou, China; 4 Department of Ophthalmology & Visual Sciences, Faculty of Medicine, The Chinese University of Hong Kong, Hong Kong, China; 5 School of Biomedical Engineering, Shenzhen Campus of Sun Yat-sen University, Shenzhen, China; 6 Ophthalmic Center, Kiang Wu Hospital, Macao SAR, Macao, China; 7 School of Computer Science and Engineering, Sun Yat-sen University, Guangzhou, China; 8 Cambridge Center for Brain Repair and MRC Mitochondrial Biology Unit, Department of Clinical Neurosciences, University of Cambridge, Cambridge, UK; 9 Cambridge Eye Unit, Addenbrooke’s Hospital, Cambridge University Hospitals, Cambridge, UK; 10 Moorfields Eye Hospital, London, UK; 11 UCL Institute of Ophthalmology, University College London, London, UK; 12 Beijing Laboratory of Brain and Cognitive Intelligence, Beijing Municipal Education Commission, Beijing, China; 13 Department of Automation and BNRist, Tsinghua University, Beijing, China; 14 Hainan Eye Hospital and Key Laboratory of Ophthalmology, Zhongshan Ophthalmic Center, Sun Yat-sen University, Haikou, China; 15 Center for Precision Medicine and Department of Genetics and Biomedical Informatics, Zhongshan School of Medicine, Sun Yat-sen University, Guangzhou, China

**Keywords:** Medical research, Signs and symptoms, Biomedical engineering, Social sciences, Health care

## Abstract

The storage of facial images in medical records poses privacy risks due to the sensitive nature of the personal biometric information that can be extracted from such images. To minimize these risks, we developed a new technology, called the digital mask (DM), which is based on three-dimensional reconstruction and deep-learning algorithms to irreversibly erase identifiable features, while retaining disease-relevant features needed for diagnosis. In a prospective clinical study to evaluate the technology for diagnosis of ocular conditions, we found very high diagnostic consistency between the use of original and reconstructed facial videos (*κ* ≥ 0.845 for strabismus, ptosis and nystagmus, and *κ* = 0.801 for thyroid-associated orbitopathy) and comparable diagnostic accuracy (*P* ≥ 0.131 for all ocular conditions tested) was observed. Identity removal validation using multiple-choice questions showed that compared to image cropping, the DM could much more effectively remove identity attributes from facial images. We further confirmed the ability of the DM to evade recognition systems using artificial intelligence-powered re-identification algorithms. Moreover, use of the DM increased the willingness of patients with ocular conditions to provide their facial images as health information during medical treatment. These results indicate the potential of the DM algorithm to protect the privacy of patients’ facial images in an era of rapid adoption of digital health technologies.

## Abstract (web summary)

A deep-learning algorithm that removes patient-identifying information from facial images, while retaining sufficient information for accurate disease diagnosis, has the potential to protect patient privacy and facilitate public acceptance of facial imaging for use in digital medicine.

## Main

Protecting the privacy of patients is central to healthcare delivery and has important ethical and medicolegal ramifications. Privacy protection has attained prominence over the past decade because of digitalization and increasingly widespread sharing of medical records and concerns about data breaches. Previous studies have explored the application of anonymization technologies for medical images. Researchers have proposed eliminating all digital imaging and communications in medicine (DICOM) metadata (such as patient name and sex)^1^, with the application of defacing or skull-stripping algorithms to face or skull regions in DICOM images^2^. From a privacy perspective, clinical data involving facial images are especially sensitive, given that facial information clearly contains biometric identifying information. It is therefore imperative to protect the facial information of healthcare users to maintain medical privacy and security; however, facial images aiming to record signs of disease, such as strabismus or nystagmus, inevitably record patients’ race, sex, age, mood and other biometric identifiers. Concerning facial images, common anonymizing methods, including blurring and cropping identifiable areas, may lose important disease-relevant information and they cannot fully evade face recognition systems^3^. An important challenge is, therefore, to separate biometric identity from medical information that can potentially be derived from facial images.

Additionally, the successful development and utility of digital health technology depends on broad participation in medical data collection and the broad participation of large populations requires trust and protection of privacy^4^; however, digital data studies based on heavy-training image sets have also raised the potential threat of misusing facial recognition technology for unintended and/or unauthorized purposes^5,6^. Due to the understandable privacy concerns of individuals, people often hesitate to share their medical data for public medical research or electronic health records, thus largely hindering the development of digital medical care. Therefore, it is necessary to update the traditional procedure used to obtain informed consent at the front end of data collection, particularly by ensuring adequate privacy protection for personal health information and somehow improving the willingness of healthcare users to engage with these emerging digital technologies.

In whole facial images, periocular biometrics is one of the most distinctive subsets of individual biometric information of an individual and it can be used to assist in building robust identity verification systems^7^. Additionally, periocular features are important signs of eye and general health. For example, periocular features, such as deep forehead wrinkles and periorbital wrinkles, are significantly associated with coronary heart disease^8^ and abnormal topological changes in eye dynamics indicate poor visual function and visual cognitive development problems^9^. This study aims to protect the biometric information of patients and focuses on four pathological ocular manifestations, namely, thyroid-associated orbitopathy (TAO), strabismus, ptosis and nystagmus, which involve more than ten abnormal behavioral phenotypes, such as eyelid retraction, overactive or underactive extraocular muscles, horizontal or vertical strabismus, changes in the double eyelid line, poor fixation and compensatory head position.

To extract these disease-relevant features but remove patient identity features from facial images of patients, we developed the DM, a new technology based on real-time three-dimensional (3D) reconstruction and deep-learning algorithms. The DM takes an original video as input and outputs a reconstructed video that contains disease information, while discarding as much of the patient’s identity as possible. The refined eye reconstruction is highlighted. Converting DM-reconstructed videos back to raw videos is impossible because most of the information necessary to recreate the original attributes has been discarded and is no longer present in the set of digital representations that constitute the mask.

To demonstrate the feasibility of the proposed DM approach, we designed a clinical trial (NCT05058599) and evaluated the consistency of the diagnoses of patients with ocular diseases from reconstructed videos and original videos. Identity removal validation was also used to show whether the DM could effectively remove personal biometric attributes. Additionally, we performed an empirical investigation of the receptiveness of patients to applying this new technology to their personal health information. Finally, we conducted an artificial intelligence (AI)-powered reidentification validation to evaluate the performance of the DM in evading recognition systems. The following results show that DM proposes a new approach to safeguarding patient privacy, provides an additional data format for privacy protection and enhances the willingness of patients to share their medical data, thereby benefiting the quickly evolving field of digital health.

## Results

### The workflow of the DM

**Fig. 1:**
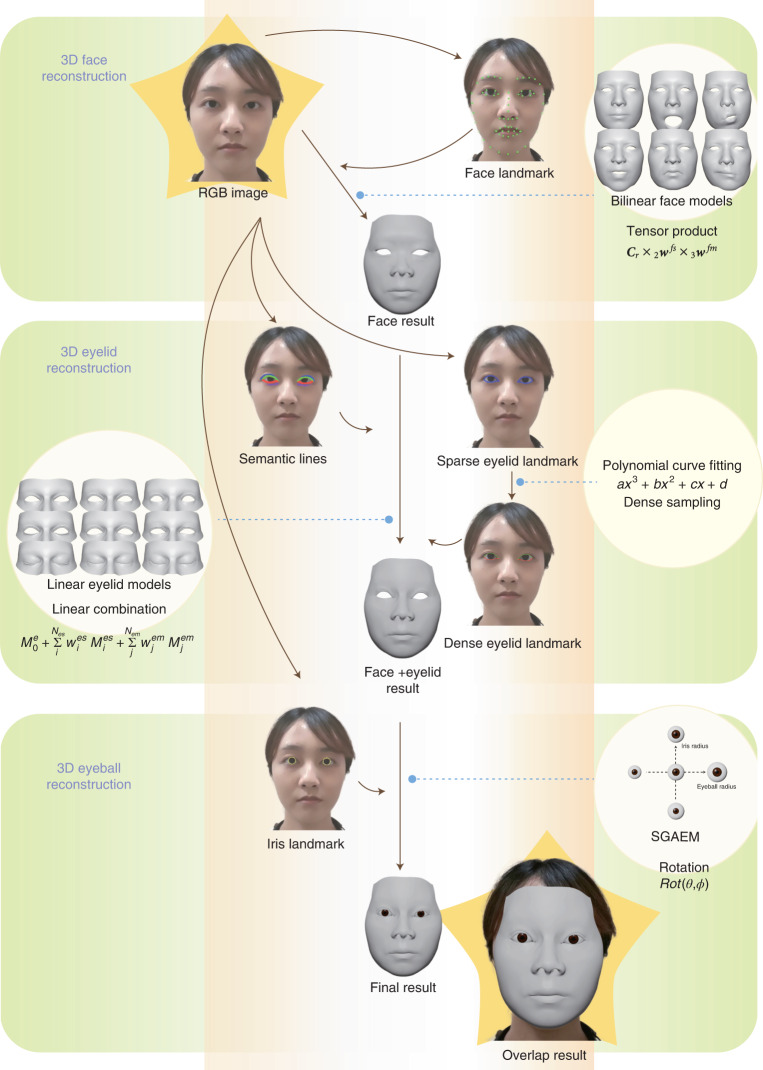
Development of the DM system. Our approach uses RGB images as input and outputs 3D reconstructed meshes. For a particular frame, the algorithm first extracts 2D face landmarks from the RGB image and fits a set of face model weights for 3D face reconstruction. Then, the algorithm extracts 2D eyelid landmarks and 2D semantic lines and fits eyelid model weights for 3D eyelid reconstruction. Finally, the algorithm extracts 2D iris landmarks and solves eyeball rotation for 3D eyeball reconstruction.

In this work, the proposed DM patient privacy protection technology was based on the complementary use of deep learning and 3D reconstruction. Deep learning achieved feature extraction from different facial parts, and 3D reconstruction automatically digitalized the shapes and motions of 3D faces, eyelids and eyeballs based on the extracted facial features (Fig. 1). Different from other face reconstruction methods^10–16^, the proposed technology focused on accurate ocular reconstruction, including both shapes and movements.

In 3D reconstruction, we used three predefined parametric models for faces, eyelids and eyeballs. The face model was mathematically a bilinear model^17^ (Methods) that represented a 3D face as a shape vector **w**^*fs*^ and a motion vector **w**^*fm*^. Given a particular **w**^*fs*^ and a **w**^*fm*^, the bilinear face model can reconstruct a particular 3D face mesh **M**^*f*^. The face model can represent the overall geometry of the face, but eye regions lack details. Since the eye regions are important for diagnosis, we used a linear eyelid model^18^ to represent detailed eye regions. Similar to the face model, given this eyelid model, a detailed eyelid **M**^*e*^ (of one eye) was represented by an eye shape vector **w**^*es*^ and an eye motion vector **w**^*em*^. To additionally reconstruct the eyeballs, we used the simplified geometry and appearance eyeball model (SGAEM), introduced in our previous study^19^. The model approximated eyeballs as spheres and used three parameters, the eyeball radius *r*_e_, the iris radius *r*_i_ and the position *p*_e_ relative to the face, to represent the static properties of an eyeball and the eyeball rotation in polar coordinates to represent eyeball motion.

Deep-learning techniques were leveraged to extract facial features that were used to infer the aforementioned model parameters to obtain the facial reconstruction results. First, a pretrained neural network was used as a face landmark detector to extract two-dimensional (2D) face landmarks **L**^*face*^ from an input red, green and blue color space (RGB) image. With the landmarks **L**^*face*^, we estimated the face pose **T** (rotation and translation), face shape vector **w**^*fs*^ and face motion vector **w**^*fm*^ by minimizing the Euclidean distance between the 2D landmarks **L**^*face*^ and the 2D projections of the corresponding points on the 3D face **M**^*f*^. Second, an eyelid landmark detector was used to extract 2D eyelid landmarks **L**^*eyelid*^ and an eyelid semantic line detector was used to extract 2D eyelid semantic lines **S**^*eyelid*^. These two detectors were also neural networks trained by deep-learning techniques. Then, we similarly estimated the eyelid shape vector **w**^*es*^ and the eyelid motion vector **w**^*em*^ by minimizing the Euclidean distance between the 2D landmarks **L**^*eyelid*^ and the projections of the corresponding points on the eyelid mesh **M**^*e*^, as well as by making the projected points on the semantic lines close to the detected semantic lines **S**^*eyelid*^ on the image. Here, semantic lines provided rich and continuous information on the eyelid area, while landmarks were robust discrete features for tracking eyelid motions. Combining these two types of features made the reconstruction more accurate and stable. Finally, for the eyeballs, we trained another neural network as an iris landmark detector to extract 2D iris landmarks **L**^*iris*^ from the input RGB image. As the eyeball radius *r*_e_, iris radius *r*_i_ and relative position *p*_e_ were invariant in a video, we predicted them in the first frame and then fixed them in the following video frames. Per-frame eyeball rotations were estimated by minimizing the Euclidean distance between the 2D landmarks **L**^*iris*^ and the projections of the corresponding points on the SGAEM. The DM included optional operations for adapting to different clinical applications, such as dealing with eye occlusion in videos recording the alternate cover test or reconstructing eyebrow movements in diagnosing ocular diseases.

### Quantitative evaluation of the DM

The feasibility of the proposed model was evaluated on a video dataset of patients in the clinical trial. From May 2020 to September 2021, 405 participants, 187 (46.2%) males, aged 4 months to 61 years, who agreed to participate in the prospective study at the Digital Mask Program either by themselves or via their legal guidance; the participants consisted of (1) 100 outpatients from strabismus departments; (2) 92 outpatients from pediatric ophthalmology departments; (3) 102 outpatients from TAO departments; and (4) 111 outpatients from oculoplastic departments (Extended Data Table 1). In total, 253 (62.47%) of the 420 patients were diagnosed with ocular diseases on the basis of face-to-face assessments of the patients’ eyes.

To evaluate the applicability of the model, different cameras, including a Nikon 3500, Huawei p30 and Sony 4k, were used for video collection according to the following standards. The whole appearances of participants were collected from a distance ranging from 33 cm to 1 m according to the specific ocular examination. These videos were taken under room illuminance ranging from 300 to 500 lx.

**Fig. 2:**
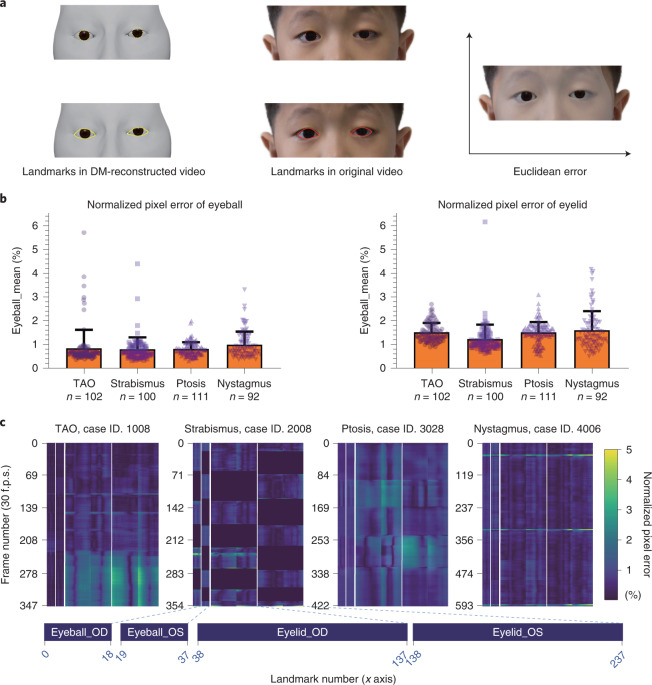
Quantitative evaluation of the digital mask. **a**, Schematic indicating how the Euclidean error was calculated. For both eyeball and eyelid reconstruction, we project the 3D points to 2D image space (yellow) and calculate the Euclidean pixel distance between them and landmarks in the original video (red). The Euclidean error was normalized by the pixel distance between the center of two eyes to exclude the influence of face size. **b**, The normalized pixel error of different ocular diseases for eyeball (left) and eyelid (right) reconstruction. Results were expressed as mean ± s.d. **c**, Heat map of the normalized pixel error for four cases. The frame number of each video (30 f.p.s.) is plotted on the *y* axis. Landmark numbers (238 in total, 38 landmarks for eyeballs and 200 landmarks for eyelids) are plotted in order on the *x* axis. Normalized pixel error (0–5%) is indicated by the collar bar at the right. The closer the color is to blue, the more accurate the performance is. OD, right eye; OS, left eye; f.p.s., frames per second.

We used the proposed DM to process all the videos and quantitatively evaluated the reconstruction performance of the DM. In the quantitative evaluation, the performance of the DM was measured by the 2D normalized pixel error, with lower numbers indicating better reconstruction performance. We first acquired the Euclidean distance between the landmarks in DM-reconstructed videos and the corresponding landmarks in original videos (Fig. 2a). The pixel errors between landmarks were then normalized by the pixel distance between the centers of the two eyes.

For the eyes of 405 patients, the average normalized pixel errors in images of patients with TAO, strabismus, ptosis and nystagmus were 0.85%, 0.81%, 0.82% and 1.00%, respectively, in eyeball reconstruction and 1.52%, 1.24%, 1.52% and 1.61%, respectively, in eyelid reconstruction (Fig. 2b). The heat map of the normalized pixel errors in images of patients with the abovementioned four diseases is shown in Fig. 2c. The normalized pixel errors remained small and stable most of the time, with slight fluctuations when the eyes were looking down, thus indicating the precise reconstruction of the DM.

### Clinical validation of DM

**Fig. 3:**
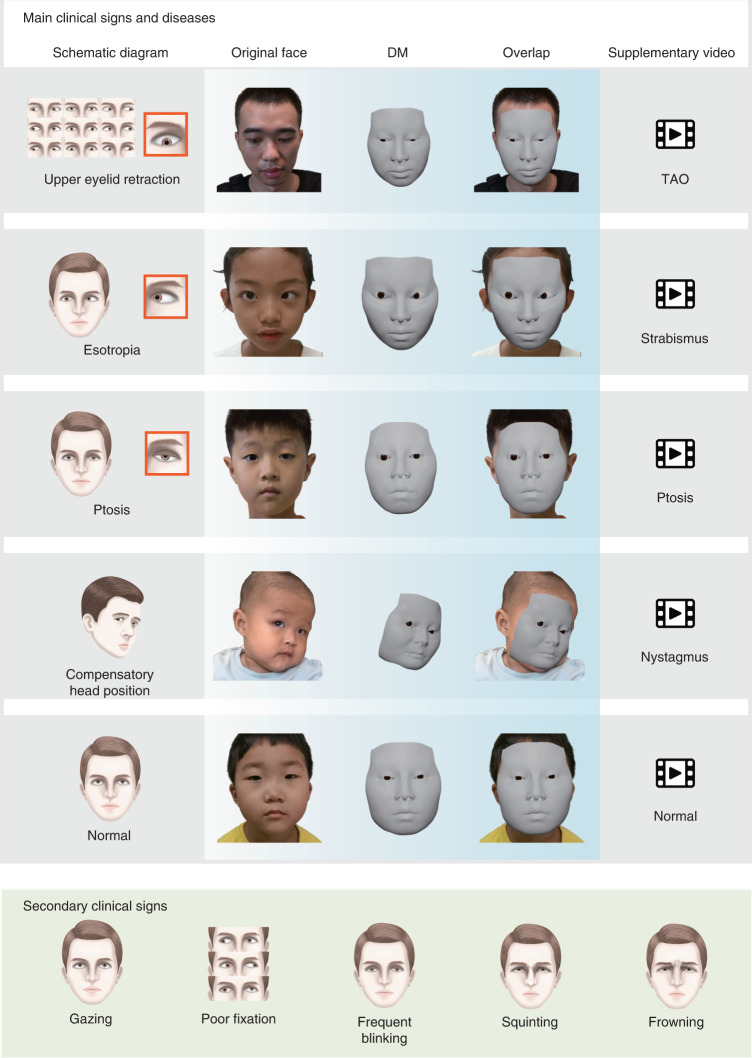
Clinical signs of the ocular diseases studied. Main clinical signs (top) for diagnosis of each ocular disease studied are shown using schematic diagram, the original facial image, the image of the DM and the overlap between the original facial image and the DM. See Supplementary Video for details. More diverse secondary clinical signs of the four diseases are shown (bottom).

**Fig. 4:**
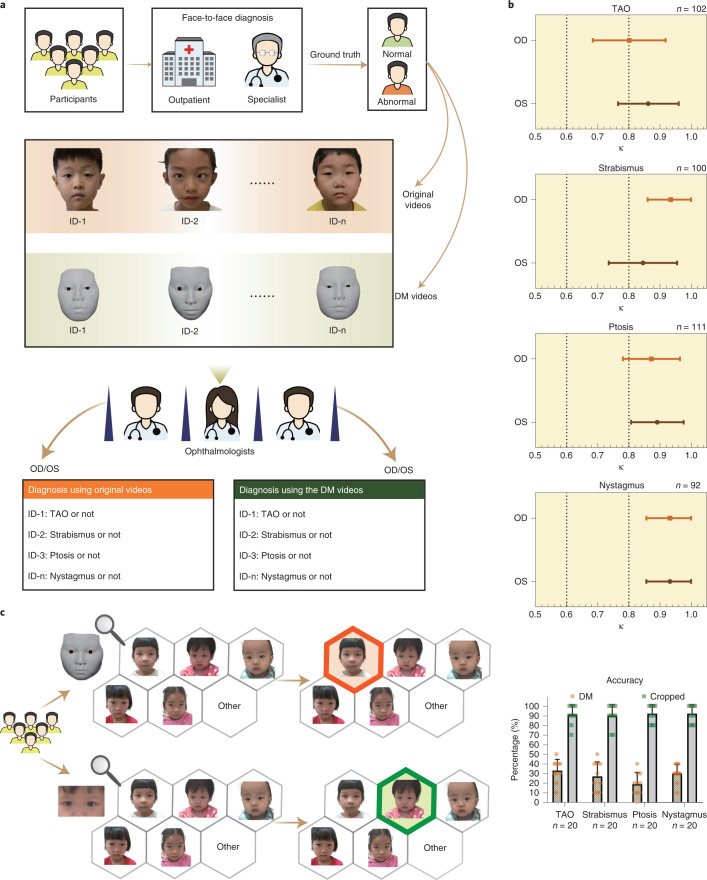
Clinical validation of the DM. **a**, Workflow of relevant diagnostic comparisons using the original videos and the corresponding DM-reconstructed videos. Participants were recruited from four outpatient departments after having been diagnosed by a specialist as having TAO, ptosis, strabismus, nystagmus or none of these. Once the participants were enrolled in the study, facial videos of appropriate ocular examinations were taken. Each video was independently used by three ophthalmologists from each of the four departments for making a diagnosis. A dichotomous diagnosis of abnormal or not was made for both the left eye and the right eye. Both the original video and DM video from the same participant were used by the same ophthalmologist for diagnosis, performed in a blinded fashion using the participant number (ID-1 to ID-n; TAO, *n* = 102; strabismus, *n* = 100; ptosis, *n* = 111; and nystagmus, *n* = 92). **b**, Line plots indicating diagnostic consistency for the indicated ocular diseases (Cohen’s *κ* ≥ 0.81 indicates perfect consistent). **c**, Left, workflow for identity removal validation in which the identity removal abilities of the DM and those of image cropping were compared. Respondents were given six options, including five facial images and an ‘other’ option. From these options, the respondents were asked to choose the original image corresponding to the DM-reconstructed image or cropped image. Red indicates an incorrect answer; green indicates a correct answer. Accuracy of identity removal validation (right). Results were expressed as mean ± s.d. Each scatter-point represents the score of one set calculated from one respondent, with ten questions per set and a total score of 100. For each disease, 20 sets of questions (10 of DM and 10 of cropped) were taken.

To evaluate the performance of the DM in clinical practice, we performed a relevant diagnostic comparison and an identity-removal validation. In the relevant diagnostic comparison, 12 ophthalmologists, 3 from each of the four departments, were invited to diagnose patients from their departments based on the DM-reconstructed videos and original videos. We evaluated the videos regarding pathological ocular manifestations that caused changes in the appearance of the eye and patients were diagnosed visually with diseases, including (1) TAO (exophthalmos, eyelid retraction and overactive or underactive extraocular muscles); (2) strabismus (horizontal or vertical strabismus and compensatory head position); (3) ptosis (drooping or lowering of the upper eyelid); and (4) nystagmus (Fig. 3 and Supplementary Video)^9^. For each eye, both the independent diagnosis from the original videos and the diagnosis from the DM-reconstructed videos were recorded and compared (Fig. 4a and Supplementary Data 1). If the two diagnoses were excellently consistent, this would suggest that the reconstruction was precise enough for use in clinical practice. Cohen’s *κ* values showed very high consistency (*κ* = 0.845–0.934 for strabismus, ptosis and nystagmus on both eyes and *κ* = 0.801 for TAO on right eyes) of the diagnoses, made by three ophthalmologists under majority rule, from original and reconstructed videos for all comparisons (Fig. 4b and Extended Data Table 2). Additionally, the accuracies of the diagnoses from the original and reconstructed videos, compared to the ground truth, were comparable for all paired comparisons (*P* = 0.131–1; Extended Data Table 3). These results indicate that the DM retains the important clinical attributes correctly and has the potential to be adopted in clinical practice.

In the identity-removal validation, we compared the identity-removal ability of the DM with that of cropping by using multiple-choice questions. Specifically, we processed the original images of the faces of the patients by using DM and cropping to generate 400 DM-reconstructed images and 400 cropped images, respectively. The selected generated images and the original images were staggered in the video time sequence. Correspondingly, we designed 800 multiple-choice questions. For the DM test, each question contained a DM-reconstructed image and five original images. For the cropping test, each question contained a cropped image and five original images. For each question, there were six options, including the five original images and an ‘other’ option. From these options, the respondents were asked to find the original image corresponding to the DM-reconstructed image or cropped image. The results showed that the accuracy rate for those taking the DM test was 27.3%; however, the accuracy for those taking the cropping was 91.3%, which was much greater than the accuracy of those taking the DM test (Fig. 4c). Both accuracies were likely overestimated because the test was conducted on the premise that the respondent knew only five people. In actual situations, the numbers of people are far higher; however, the results still demonstrate that the DM can effectively remove patient identity attributes and protect patient privacy, especially compared to cropping.

**Fig. 5:**
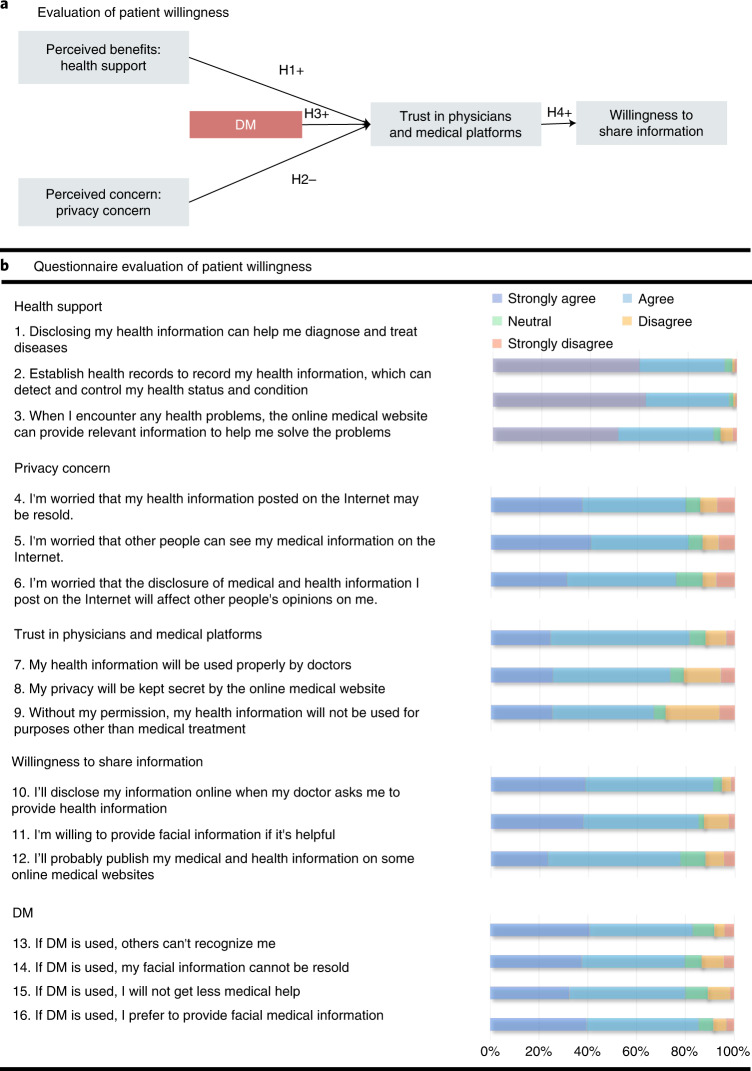
Empirical investigation of the willingness of patients to share personal health information. **a**, Schematic of the hypothesis. Patients’ trust in physicians and medical platforms was hypothesized to be positively affected by perceived benefits, such as health support of digital health information (H1) and negatively affected by perceived concerns, such as privacy concerns (H2). The DM was hypothesized to have a positive impact on such trust (H3) and further improved patients’ willingness to share information (H4). **b**, Questionnaire results. In the questionnaire, 16 questions were designed with respect to the five hypotheses; the responses were further measured using five-point scales ranging from ‘strongly disagree’ to ‘strongly agree’. The percentages of the responses to each item are shown (right).

In addition, to evaluate the willingness of patients to share their eye and facial images during the application of DM, we performed an empirical investigation. 3D reconstruction software was developed to which users could provide their videos anonymously. The videos were then automatically processed by the DM and delivered to clinicians. Clinicians were only allowed to watch the DM-reconstructed videos for diagnosis (Supplementary Video) and the diagnoses were fed back to the users. A total of 317 outpatients, randomly selected via clinics, agreed to participate in the empirical investigation. During the investigation, the participants were asked to watch uploaded videos and the corresponding reconstructed videos processed by the DM using the software. The patients then completed a questionnaire to investigate their willingness to use DM at the end of the investigation (Fig. 5a). Among the respondents, 161 were males (50.7%). By age group, the highest proportion of respondents was in the 20–30-year group. Most of the respondents had university degrees (82.3%) and had used smartphones for more than 7 years (73.8%). In addition, in the questionnaire, regarding five hypotheses, 16 questions were designed from five aspects, including health support, privacy concerns, trust in physicians and medical platforms, willingness to share information and the influences of DM (Fig. 5b). The Kaiser–Meyer–Olkin measure of sampling adequacy and Cronbach α values for each component were larger than 0.617 and 0.718, respectively, thus supporting the reliability and validity of each question in the research design. Approximately 80% of the participants agreed that they had privacy concerns. Among the participants who had a disease with facial signs, more than 81.4% had privacy concerns, compared to more than 74.4% of participants without facial signs. Furthermore, we assessed the significance of the influence of the major aspects.

As shown in Extended Data Table 4, perceived benefits, such as health support of digital health information, positively affected patients’ trust in physicians and medical platforms with respect to digital health (*β* = 0.465, *P* < 0.001). In contrast, perceived concerns, such as privacy concerns, negatively affected patients’ trust in physicians with respect to digital health (*β* = −0.158, *P* = 0.005). The hypothesis that the DM had a positive impact on such trust was supported (*β* = 0.348, *P* < 0.001), thereby further improved the patients’ willingness to share information (*β* = 0.503, *P* < 0.001). The questionnaire details of each patient are included in Supplementary Data 2.

### AI-powered re-identification validation of the DM

**Fig. 6:**
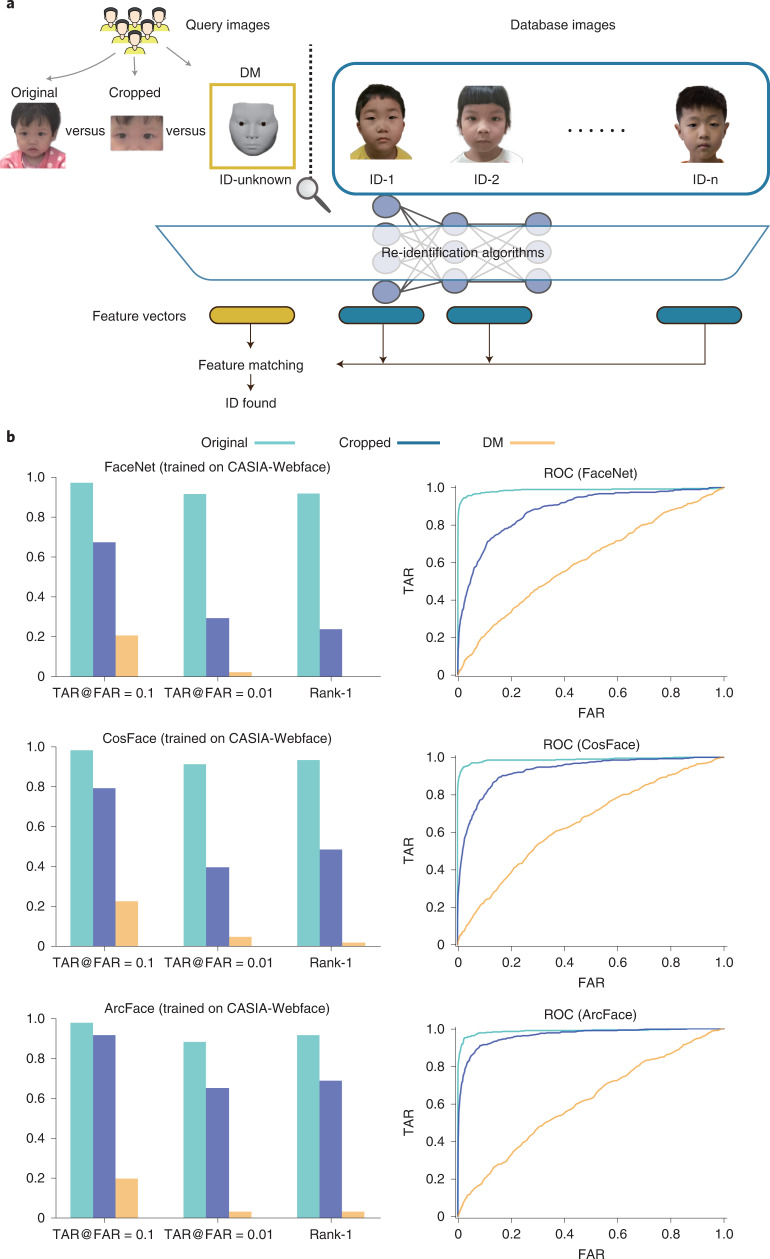
Validation of the DM using AI-powered re-identification algorithms. **a**, Study workflow. The re-identification algorithms were used to find the ID of the patient from a database of 405 patients when given the original image, a cropped image or the DM-reconstructed image of a patient as a query image. **b**, Performance of the three re-identification algorithms tested, as assessed by TAR@FAR = 0.1, TAR@FAR = 0.01, Rank-1 (left) and ROC curves (right). The re-identification algorithms were trained on the CASIA-Webface dataset. TAR@FAR = X indicates the TAR when the FAR equals X. Rank-1 is the probability that the similarity score of the same identity ranks first among all the identities. Lower values of TAR@FAR = 0.1, TAR@FAR = 0.01 and Rank-1 indicate weaker performance of the re-identification algorithm and better performance of the privacy protection technology. TAR = TP/(TP + FN); FAR = FP/(FP + TN). TP, true positive; FP, false positive; TN, true negative; FN, false negative.

To evaluate the performance of the DM in evading recognition systems, we performed an AI-powered reidentification validation (Fig. 6a). In the validation, we conducted face recognition attacks by using three well-known deep-learning systems, namely, FaceNet^20^, CosFace^21^ and ArcFace^22^. All the systems were trained on the CASIA-WebFace Dataset^23^, which contains 494,414 face images of 10,575 real identities collected from the web. Using 405 patient videos, we randomly selected two frames in each video; one of the frames was used as the query image and the other was used as the database image. We processed 405 original query images to further generate 405 cropped query images and 405 DM-reconstructed query images. For the test, given a query image (original images, cropped images or DM-reconstructed images), the face recognition system (FaceNet, CosFace or ArcFace) was asked to match the image with database images of 405 patients. We used the area under the receiver operating characteristic curve (AUC), TAR@FAR = 0.1 (TAR, true accept rate; FAR, false accept rate), TAR@FAR = 0.01 and Rank-1 to evaluate the face recognition performance. The lower values of TAR@FAR = 0.1, TAR@FAR = 0.01 and Rank-1 indicate the weaker performance of the face recognition system and the greater performance of the privacy protection technology. As shown in Fig. 6b and Extended Data Table 5, the results on all the measurements show that taking original images as the query images, it was easy for face recognition systems to match the correct identity. When taking cropped images as the query images, the metrics had limited degradation. When using the DM, the performance of face recognition was significantly degraded. Rank-1 was <0.02 for all three systems, indicating that the systems had a very low possibility of identifying the correct identity with the DM-reconstructed images. Meanwhile, the receiver operating characteristic (ROC) curves of using DM-reconstructed images were close to *y* = *x* for all three systems, indicating that it was impossible to keep high TAR with low FAR. These results show the superiority in terms of privacy protection of our DM technique.

## Discussion

In this study, we developed and validated a new technology called DM, which is based on real-time 3D reconstruction and deep learning, to retain the clinical attributes contained in patient videos, while minimizing access to nonessential biometric information for added personal privacy in clinical practice. Experimental results support that with the DM, examination videos of patients with manifestations of ocular disease can be precisely reconstructed from 2D videos containing original faces. A clinical diagnosis comparison showed that ophthalmologists achieved high consistency in reaching the same diagnosis when using the original videos and the corresponding DM-reconstructed videos. This new technology could effectively remove identity attributes and was positively accepted by patients with ocular diseases, who expressed an increasing willingness to share their personal information and have it stored digitally with this added layer of biometric protection.

It is notable that the DM offers a pragmatic approach to safeguarding patient privacy and data utilization in both research and clinical settings; patient privacy and data utilization are frequently cited as concerns by patients worried about data breaches. Compared to rather crude but still widely used options, such as covering identifiable areas with very large bars or cropping these areas out altogether^3^, the DM is a much more sophisticated tool for anonymizing facial images. Even next-generation privacy-protection techniques, such as federated learning and homomorphic encryption, do not safeguard privacy well and crucially, these techniques are vulnerable to model inversion or reconstruction attacks^24^. The DM selects relevant features for reconstruction, but it is impossible to reconstruct original data particularly relevant to patient identification. Furthermore, compared with other face-swapping technologies, the DM can obtain quantitative parameters (such as the degree of eyeball rotation, eyelid shape parameters, blinking rate and rotation frequency), which might prove essential in the future for intelligent diagnosing disease or studying the relationships between diseases and certain facial characteristics.

In addition to its potential utilization in research and routine clinical practice, the DM can be applied to telemedicine, including online automatic diagnosis and patient triage for more efficient healthcare delivery^25^. The wider adoption of digital medicine, partly prompted by the ongoing COVID-19 pandemic, will require that the barriers to privacy protection be overcome and an important step is removing biometric data that are not essential for healthcare delivery. The DM can encrypt data before they are submitted to the cloud, thereby allowing clinicians or AI algorithms to review the reconstructed data and removing concerns of patients whose medical records contain sensitive biometric data^26^.

However, ‘protecting privacy’ does not equate to ‘absolute removal of identity characteristics.’ According to the Health Insurance Portability and Accountability Act Privacy Rule, protecting patient privacy refers to reducing the identification risk of health information^27^. One of the most important principles is balancing disclosure risk against data utility. Therefore, the purpose of this study is to provide an approach to health information disclosure that de-identifies protected health information as much as possible, without compromising the need for the clinician to reach a diagnosis.

The study has several limitations. First, the reconstruction of conjunctival hyperemia, eyelid edema and abnormal growth of tissues, such as ocular tumors, remains challenging because of insufficient model capacity. Model-based 3D reconstruction assumes that the target lies in the linear space spanned based on a set of prepared models; however, it is difficult to cover all shapes in the aforementioned cases because shapes differ significantly from person to person. We intend to improve the DM by including a sufficiently large sample of abnormal cases for more detailed analysis or constructing an extra submodel on top of the existing model in the next research step. Second, this paper has demonstrated that the DM can protect re-identification from images but it may not work under certain circumstances if the video of the patient is exposed. We are currently extending our work to deal with video protection and circumvent this possible weakness. Third, the potential risk that the DM might be attacked still remains, as it might be abused to develop targeted attack algorithms; however, this risk can be mitigated by formulating relevant rules in the future.

In conclusion, we demonstrate the effectiveness of the DM in enhancing patient data privacy by making use of deep learning and real-time 3D reconstruction and notably, we demonstrate the DM’s acceptability to healthcare users. Future work is necessary to further evaluate the applicability of DM in a wider variety of clinical settings as the requirements for de-identification will vary according to the type of imaging dataset used.

## Methods

### Ethical approval

The research protocol and ethical review of this study were approved by the Institutional Review Board/Ethics Committee of the Zhongshan Ophthalmic Center. The clinical study protocol is shown in the Supplementary Note. Consent was obtained from all individuals whose images are shown in figures or the video for publication of these images. Informed consent was obtained from at least one legal guardian of each infant and the tenets of the Declaration of Helsinki were followed throughout this study. The trial in this study was registered with the Clinical Research Internal Management System of Zhongshan Ophthalmic Center and retrospectively registered at ClinicalTrials.gov (NCT05058599).

### DM technique

Our reconstruction method consisted of three main stages: face reconstruction, eyelid reconstruction and eyeball reconstruction. At each stage, a unique detector was used to extract relevant features (the face landmarks **L**^*face*^ at the first stage, the eyelid landmarks **L**^*eyelid*^ and eyelid semantic lines **S**^*eyelid*^ at the second stage and the iris landmarks **L**^*iris*^ at the last stage). All detectors were neural networks based on deep-learning techniques. After acquiring the features, the corresponding model parameters were optimized to fit these features. The details of each stage are described below.

### Face reconstruction

As we use a bilinear model^17^, we generated a 3D face ${{{\boldsymbol{M}}}}^{\,f} \in {\Bbb R}^{3N_F}$ using a shape vector ${{{\boldsymbol{w}}}}^{\,fs} \in {\Bbb R}^{N_{fs}}$ and a set of motion vectors ${{{\boldsymbol{w}}}}^{\,fm} \in {\Bbb R}^{N_{fm}}$:

1 $${{{{\boldsymbol{M}}}}^{\,f} = {{{\boldsymbol{C}}}}_r \times _2{{{\boldsymbol{w}}}}^{\,fs} \times _3{{{\boldsymbol{w}}}}^{\,fm}}$$

where ${{{\boldsymbol{C}}}}_r \in {\Bbb R}^{3N_F \times N_{fs} \times N_{fm}}$ is a pre-defined core tensor that stores 3D vertex positions of faces covering the major variations in shape and motion; ×_2_ and ×_3_ are the tensor product operations on the second dimension and third dimension, respectively; *N*_F_ is the number of 3D face vertices; and *N*_fs_ and *N*_fm_ are the dimensions of the shape vector and motion vector, respectively.

Given the face landmarks ${{{\boldsymbol{L}}}}^{face} \in {\Bbb R}^{2N_{LF}}$ on a video frame of a patient, we reconstructed a 3D face of the patient by solving an optimization problem; minimizing the landmark registration error *E*_face_ by searching for the optimal parameters **w**^*fs*^, **w**^*fm*^, **R** and **t**:

2 $$E_{face}\left( {{{{\boldsymbol{w}}}}^{\,fs},{{{\boldsymbol{w}}}}^{\,fm},{{{\boldsymbol{R}}}},{{{\boldsymbol{t}}}};{{{\boldsymbol{C}}}}_r,{{{\boldsymbol{L}}}}^{face}} \right) = \mathop {\sum}\limits_i^{N_{LF}} {\left\| {{{{\boldsymbol{L}}}}_i^{face} - {{{\mathrm{{\Pi}}}}}\left( {{{{\boldsymbol{M}}}}^{\,f\prime }_{c_{face}(i)}} \right)} \right\|_2^2}$$

3 $$\begin{array}{*{20}{c}} {{{{\boldsymbol{M}}}}^{\,f\prime } = \boldsymbol{R}{{{\boldsymbol{M}}}}^{\,f} + \boldsymbol{t}} \end{array}$$

where **R** ∈ *SO*(3) and ${{{\boldsymbol{t}}}} \in {\Bbb R}^3$ denote the rotation and translation of a 3D mesh, respectively; ∏(·) is a projection function that projects 3D points to 2D points; *c*_face_(*i*) represents the corresponding face index for the *i*th face landmark, which is predefined manually; and *N*_LF_ is the number of face landmarks.

Note that **w**^*fs*^ was estimated based on only the first frame and then fixed for the following frames. Therefore, for the following frames, the objective function was slightly simplified to

4 $$E_{face}\left( {{{{\boldsymbol{w}}}}^{\,fm},{{{\boldsymbol{R}}}},{{{\boldsymbol{t}}}};{{{\boldsymbol{w}}}}^{\,fs},{{{\boldsymbol{C}}}}_r,{{{\boldsymbol{L}}}}^{face}} \right) = \mathop {\sum}\limits_i^{N_{LF}} {\left\| {{{{\boldsymbol{L}}}}_i^{face} - {{{\mathrm{{\Pi}}}}}\left( {{{{\boldsymbol{M}}}}^{\,f\prime }_{c_{face}(i)}} \right)} \right\|_2^2}$$

### Eyelid reconstruction

Similar to the bilinear face model, our eyelid model^18^ contained a set of shape vectors ${{{\boldsymbol{w}}}}^{es} \in {\Bbb R}^{N_{es}}$ and a set of motion vectors ${{{\boldsymbol{w}}}}^{em} \in {\Bbb R}^{N_{em}}$. *N*_es_ and *N*_em_ are the dimensions of the shape vector and motion vector, respectively. Given our parametric eyelid model, a particular 3D eye region ${{{\boldsymbol{M}}}}^e \in {\Bbb R}^{3N_D}$ was reconstructed as follows:

5 $${{{\boldsymbol{M}}}}^e = {{{\boldsymbol{M}}}}_0^e + \mathop {\sum}\limits_i^{N_{es}} {w_i^{es}{{{\boldsymbol{M}}}}_i^{es}} + \mathop {\sum}\limits_j^{N_{em}} {w_j^{em}{{{\boldsymbol{M}}}}_j^{em}}$$

where ${{{\boldsymbol{M}}}}_0^e \in {\Bbb R}^{3N_D}$ is the template eyelid geometry model; ${{{\boldsymbol{M}}}}^{es} \in {\Bbb R}^{N_{es} \times 3N_D}$ and ${{{\boldsymbol{M}}}}^{em} \in {\Bbb R}^{N_{em} \times 3N_D}$ are also predefined and represent the basis geometry changes for shape and motion, respectively; and *N*_D_ is the number of 3D eyelid vertices.

Before reconstruction, we first fitted two polynomial curves for the upper eyelid and the lower eyelid according to the detected landmarks ${{{\boldsymbol{L}}}}^{eyelid} \in {\Bbb R}^{2N_{Ld}}$. Specifically, we fit cubic polynomial curves:

6 $$\begin{array}{*{20}{c}} {y = ax^3 + bx^2 + cx + d} \end{array}$$

by solving a least-squares problem:

7 $$\begin{array}{*{20}{c}} {\left( {\begin{array}{*{20}{c}} 1 & {x_1} & {x_1^2} & {x_1^3} \\ 1 & {x_2} & {x_2^2} & {x_2^3} \\ \vdots & \vdots & \vdots & \vdots \\ 1 & {x_{N_{Ld}}} & {x_{N_{Ld}}^2} & {x_{N_{Ld}}^3} \end{array}} \right)\left( {\begin{array}{*{20}{c}} d \\ c \\ b \\ a \end{array}} \right) = \left( {\begin{array}{*{20}{c}} {y_1} \\ {y_2} \\ \vdots \\ {y_{N_{Ld}}} \end{array}} \right)} \end{array}$$

*x* and *y* denote the 2D coordinates of a point on 2D image. Then, we applied dense sampling to acquire dense landmarks ${{{\boldsymbol{L}}}}^{dense} \in {\Bbb R}^{2N_{LD}}$ by uniform sampling $x^{dense} = \{ x_1^{dense},x_2^{dense}, \cdots ,x_{N_{LD}}^{dense}\}$.

8 $$\begin{array}{ll}{\boldsymbol{L}}_i^{dense} &= \left( {x_i^{dense},y_i^{dense}} \right)\\ &= \left( {x_i^{dense},a\left( {x_i^{dense}} \right)^3 + b\left( {x_i^{dense}} \right)^2 + c\left( {x_i^{dense}} \right) + d} \right)\end{array}$$

where *N*_*Ld*_ is the number of detected eyelid landmarks and *N*_LD_ is the number of dense landmarks.

For continuous features, the four detected semantic lines (representing the double-fold, the upper eyelid, the lower eyelid and the lower boundary of the bulge) **S**^*eyelid*^ are irregular curves defined on the 2D image space, thus indicating the positions of different parts of the eyelid.

Integrating both discrete features and continuous features, we solved the following energy function to search for the optimal **w**^*es*^ and **w**^*em*^:

9 $$E_{eyelid}\left( {{{{\boldsymbol{w}}}}^{es},{{{\boldsymbol{w}}}}^{em};{{{\boldsymbol{R}}}},{{{\boldsymbol{t}}}},{{{\boldsymbol{M}}}}_0^e,{{{\boldsymbol{M}}}}^{es},{{{\boldsymbol{M}}}}^{em},{{{\boldsymbol{L}}}}^{dense}} \right) = \mathop {\sum}\limits_i^{N_{LD}} {\left\| {{{{\boldsymbol{L}}}}_i^{dense} - {{{\mathrm{{\Pi}}}}}\left( {{{{\boldsymbol{M}}}}^{e\prime }_{c_{eyelid}\left( i \right)}} \right)} \right\|_2^2}$$

10 $$E_{sl}\left( {{{{\boldsymbol{w}}}}^{es},{{{\boldsymbol{w}}}}^{em};{{{\boldsymbol{R}}}},{{{\boldsymbol{t}}}},{{{\boldsymbol{M}}}}_0^e,{{{\boldsymbol{M}}}}^{es},{{{\boldsymbol{M}}}}^{em},S^{eyelid}} \right) = \mathop {\sum}\limits_k^{N_{sl}} {\mathop {\sum}\limits_{j \in v_{sl}\left( k \right)} {dis\left( {{{{\mathrm{{\Pi}}}}}\left( {{{{\boldsymbol{M}}}}^{e\prime} _j} \right),S_k^{eyelid}} \right)} } ^2$$

11 $$\begin{array}{*{20}{c}} {{{{\boldsymbol{M}}}}^{e\prime } = \boldsymbol{R}{{{\boldsymbol{M}}}}^e + \boldsymbol{t}} \end{array}$$

where *c*_eyelid_(*i*) represents the corresponding vertex index for the *i*th eyelid landmark, which is also manually predefined in advance. *v*_sl_(*k*) represents a set of vertex indices belonging to the *k*th semantic line. *dis*(·,·) is the distance between a point and the closest point on a line. *N*_sl_ is the number of semantic lines, which is four in this paper. **R** and **t** are calculated at the face reconstruction stage.

Similar to the face reconstruction, **w**^*es*^ was determined in the first frame. In the following frames, the objective functions were changed to

12 $$E_{eyelid}\left( {{{\boldsymbol{w}}}}^{em};{{{{\boldsymbol{w}}}}^{es},{{{\boldsymbol{R}}}},{{{\boldsymbol{t}}}},{{{\boldsymbol{M}}}}_0^e,{{{\boldsymbol{M}}}}^{es},{{{\boldsymbol{M}}}}^{em},{{{\boldsymbol{L}}}}^{dense}} \right) = \mathop {\sum}\limits_i^{N_{LD}} {\left\| {{{{\boldsymbol{L}}}}_i^{dense} - {{{\mathrm{{\Pi}}}}}\left( {{{{\boldsymbol{M}}}}^{e\prime }_{c_{eyelid}\left( i \right)}} \right)} \right\|_2^2}$$

13 $$E_{sl}\left( {{{\boldsymbol{w}}}}^{em};{{{{\boldsymbol{w}}}}^{es},{{{\boldsymbol{R}}}},{{{\boldsymbol{t}}}},{{{\boldsymbol{M}}}}_0^e,{{{\boldsymbol{M}}}}^{es},{{{\boldsymbol{M}}}}^{em},S^{eyelid}} \right) = \mathop {\sum}\limits_k^{N_{sl}} {\mathop {\sum}\limits_{j \in v_{sl}\left( k \right)} {dis\left( {{{{\mathrm{{\Pi}}}}}\left( {{{{\boldsymbol{M}}}}^{e\prime} _j} \right),S_k^{eyelid}} \right)} } ^2$$

### Eyeball reconstruction

Our SGAEM model^19^ represented a 3D eyeball ${{{\boldsymbol{B}}}} \in {\Bbb R}^{3N_B}$ based on the eyeball radius *r*_e_ and the iris radius *r*_i_. *N*_B_ is the number of 3D eyeball vertices.

14 $${{{\boldsymbol{B}}}}= SGAEM\left( {r_e,r_i} \right)$$

The position of the eyeball relative to the face ${{{\boldsymbol{p}}}}_e \in {\Bbb R}^3$ is also needed to be determined for reconstruction. Here, certain prior knowledge is used to estimate the three parameters (*r*_e_, *r*_i_ and *p*_e_) in the first frame and then fixed in the following frames by minimizing the following objective function:

15 $$E_{eyeball}\left( {\theta ,\phi ;{{{\boldsymbol{R}}}},{{{\boldsymbol{t}}}},{{{\boldsymbol{L}}}}^{iris},r_e,r_i,{{{\boldsymbol{p}}}}_e} \right) = \mathop {\sum}\limits_i^{N_{LB}} {\left\| {{{{\boldsymbol{L}}}}_i^{iris} - {{{\mathrm{{\Pi}}}}}\left( {{{{\boldsymbol{B}}}}^{\prime} _{c_{iris}\left( i \right)}} \right)} \right\|_2^2}$$

16 $${{{{\boldsymbol{B}}}}^{\prime} = \boldsymbol{R}\left( {Rot\left( {\theta ,\phi } \right){{{\boldsymbol{B}}}} + {{{\boldsymbol{p}}}}_e} \right) + \boldsymbol{t}}$$

where *θ* and *ϕ* are the Euler angles of eyeball rotation. *Rot*(·,·) is a function that converts *θ* and *ϕ* into a rotation matrix. *c*_iris_(*i*) represents the corresponding vertex index for the *i*th iris landmark, which is also predefined in advance. *N*_LB_ is the number of iris landmarks. ***R*** and ***t*** are calculated at the face reconstruction stage.

### Sequence consistency

To maintain consistency between successive frames, the following smoothing terms were also considered for the above objective functions:

17 $$E_{smooth1} = \lambda _{fm}\left\| {{{{\boldsymbol{w}}}}^{{\,fm}} - {{{\boldsymbol{w}}}}_{prev}^{\,fm}} \right\|_2^2 + \lambda _R\left\| {{{{\boldsymbol{R}}}} - {{{\boldsymbol{R}}}}_{prev}} \right\|_2^2 + \lambda _t\left\| {{{{\boldsymbol{t}}}} - {{{\boldsymbol{t}}}}_{{\it{prev}}}} \right\|_2^2$$

18 $$\begin{array}{*{20}{c}} {E_{smooth2} = \lambda _{em}\left\| {{{{\boldsymbol{w}}}}^{em} - {{{\boldsymbol{w}}}}_{prev}^{em}} \right\|_2^2} \end{array}$$

19 $$\begin{array}{*{20}{c}} {E_{smooth3} = \lambda _\theta \left\| {\theta - \theta _{prev}} \right\|_2^2 + \lambda _\phi \left\| {\phi - \phi _{prev}} \right\|_2^2} \end{array}$$

where subscript *prev* represents the parameter at the previous frame.

Finally, the objective function for the three stages becomes

20 $$\begin{array}{*{20}{c}} {E_1 = E_{face} + E_{smooth1}} \end{array}$$

21 $$\begin{array}{*{20}{c}} {E_2 = E_{eyelid} + E_{sl} + E_{smooth2}} \end{array}$$

22 $$\begin{array}{*{20}{c}} {E_3 = E_{eyeball} + E_{smooth3}} \end{array}$$

The Gauss–Newton method was adopted to solve the nonlinear least-squares problem to minimize each objective function.

### Network training

We introduced how to train the networks of the three landmark detectors (face, eyelid and iris landmark detectors) and the eyelid semantic line detector (Supplementary Table 1). The network architecture for the three landmark detectors follows HRNet^28^. For the face detector, we used both the 300W^29^ and WFLW^30^ to train the network. For the eyelid and iris landmark detectors, we used UnityEyes^31^ to synthesize 20,000 images with groundtruth landmark positions in the training. The network architecture for the eyelid semantic line detector follows HED^32^ and we used the data in our previous work^18^ to train it. After all these networks were trained, we further used our own collected 775 patient portraits to fine-tune the networks, making the networks better able to handle the data of real patients. Specifically, we split 75 patient portraits from the fine-tuning dataset for validation and used the remaining 700 portraits for fine-tuning. The characteristics of the training dataset are shown in Extended Data Table 6.

### Deformation transfer for eyebrow movements

Although the linear eyelid model provided sufficient eyelid variations, it included no degrees of freedom for motion in the eyebrow region. Reconstructing the eyebrow motions of patients would help in diagnosing TAO. To perform such a reconstruction, a deformation transfer method was applied, as described below.

We defined two semantic regions on both the face and eyelid models and by assuming that the region on the face model influences the corresponding semantic region on the eyelid model, the influence of the face vertex on the eyelid vertex could be estimated based on the influence weights *w*_*i,j*_:

23 $$\begin{array}{*{20}{c}}{w_{i,j} = exp\left( { - \frac{{\left\| {{{{\boldsymbol{v}}}}_i^{\,f} - {{{\boldsymbol{v}}}}_j^e} \right\|_2^2}}{{2r^2}}} \right)} \end{array}$$

where ${{{\boldsymbol{v}}}}_i^{\,f}$ and ${{{\boldsymbol{v}}}}_j^e$ represent the *i*th face vertex and *j*th eyelid vertex, respectively and *r* is the influence radius. With *w*_*i,j*_, each eyelid vertex ${{{\boldsymbol{v}}}}_j^e$ can be deformed together with the motion of the face vertices as follows:

24 $$\begin{array}{*{20}{c}} {{{{\boldsymbol{v}}}}_{j,t}^e = {{{\boldsymbol{v}}}}_{j,t - 1}^e + \mathop {\sum}\limits_{i \in {{{\mathcal{N}}}}\left( j \right)} {\frac{{w_{i,j}}}{N}\left( {{{{\boldsymbol{v}}}}_{i,t}^{\,f} - {{{\boldsymbol{v}}}}_{i,t - 1}^{\,f}} \right)} } \end{array}$$

where *t* and *t* − 1 represent the time index of the current frame and the previous frame, respectively. ${{{\mathcal{N}}}}(j)$ is a set of face vertex indices related to ${{{\boldsymbol{v}}}}_j^e$ and *N* is the number of related vertices. Notice that all the vertices were in the local coordinate system, which removes the influence of global rotation ***R*** and global translation ***t***.

### Definitions of pathological ocular manifestations for the clinical evaluation

Ptosis is defined as the upper eyelid falling to a position that is lower than normal (typically 1.0–2.0 mm below the superior corneoscleral limbus)^33^. The palpebral fissure distance is often evaluated by guiding the patient’s eye fixation to a distant target^34^. The frontalis muscle, levator palpebrae muscle and orbicular muscle are analyzed based on a series of movement guidelines to preliminarily explore the cause of ptosis; these movement guidelines include having the patient gaze upwards and downwards, maintain an upwards gaze for 1 min and close his or her eyes tightly shut^35^. Additionally, the presence of Brown’s ocular movements and jaw motion are all provided to aid in diagnosing ptosis^36^.

Strabismus is characterized as the eyes not properly aligning with each other when looking at an object. The cover test and alternate cover test are used in diagnosing strabismus^37^. Because most people have exotropia but do not need treatment, we excluded exotropia when determining the diagnosis of strabismus. The test allows wearing glasses, especially in the case of patients with accommodative esotropia.

TAO is diagnosed by positive responses of eyelid retraction and at least two of the following four sets of findings: chemosis or eyelid edema, lid lag or restrictive lagophthalmos^38,39^.

Nystagmus is characterized as the eyes moving rapidly and uncontrollably; this movement can be observed and diagnosed during eye movement recording^40^. Additionally, compensatory head position and median zone are important features of nystagmus^41^.

### Statistical analysis

In the sample size estimate of the clinical trial, the power was set at 0.9, the significance level was 0.025 and a one-sided test was used. Assuming k1 = 0.85 and k0 = 0.6, the probabilities of abnormal findings were 0.3 to 0.7 and the sample size for each disease was at least 82 estimated using the irr package in R 4.1.1 (R Project for Statistical Computing).

Our quantitative evaluation was based on the 2D pixel distance between the detected 2D landmarks and projected 2D positions of the 3D points on the reconstructed face. To exclude the influence of face size, we evaluated our method using the normalized pixel distance rather than the absolute pixel distance.

To acquire the normalized pixel distance, we calculated the absolute pixel distance first:

25 $$\begin{array}{*{20}{c}} {D_i^{abs} = \left\| {{{{\boldsymbol{L}}}}_i - {{{\mathrm{{\Pi}}}}}\left( {{{{\boldsymbol{V}}}}_i} \right)} \right\|_2} \end{array}$$

where *L*_*i*_ is the *i*th 2D landmark and *V*_*i*_ is the *i*th 3D point.

Then, we calculated the absolute pixel distance between the two eyes:

26 $$\begin{array}{*{20}{c}} {D^{eye} = \left\| {{{{\boldsymbol{C}}}}^{left} - {{{\boldsymbol{C}}}}^{right}} \right\|_2} \end{array}$$

where **C**^*left*^ and **C**^*right*^ are the center positions of the left and right eyes, respectively.

Finally, we normalized the pixel distance between landmarks according to the distance between the two eyes, that is,

27 $$\begin{array}{*{20}{c}} {D_i^{norm} = \frac{{D_i^{abs}}}{{D^{eye}}}} \end{array}$$

To further validate the reconstruction, the maximum normalized error and average normalized error were defined as

28 $$\begin{array}{*{20}{c}} {MRE = max\left\{ {D_1^{norm},D_2^{norm}, \ldots ,D_N^{norm}} \right\}} \end{array}$$

29 $$\begin{array}{*{20}{c}} {ARE = \frac{{\mathop {\sum}\nolimits_{i = 1}^N {D_i^{norm}} }}{N}} \end{array}$$

Generally, *n* = 38 for eyeball validation and *n* = 200 for eyelid validation, but we excluded some (landmark, point) pairs when they were occluded, especially in the strabismus dataset.

In the clinical validation, the characteristics of the participants were described as the frequency (proportion) for categorical variables and the median (IQR) for continuous variables due to nonnormal distributions. Cohen’s *κ* statistics were used to evaluate the diagnostic consistency in the relevant diagnostic comparison. Kappa was interpreted as recommended by Landis and Koch, where *κ* ≤ 0.00 is considered as poor, 0.00–0.20 as slight, 0.21–0.40 as fair, 0.41–0.60 as moderate, 0.61–0.80 as substantial and ≥0.81 almost perfect^42^. In addition, based on the groundtruth, we measured the accuracies of diagnoses from the original videos and diagnoses from the reconstructed videos and compared them using the McNemar test. In the empirical investigation, principal-component analysis was used to generate five factors from the 16 questions in the questionnaire. The Kaiser–Meyer–Olkin measure of the sampling adequacy and Cronbach’s *α* for each component were used to evaluate the reliability and validity of each question. Linear regression was used to measure the associations between components.

In the AI-powered re-identification validation, we used AUC, TAR@FAR = 0.1, TAR@FAR = 0.01 and Rank-1 to evaluate the performance of face-recognition systems. The TAR is the proportion of authorized people who the system correctly accepts and is defined as

30 $$\begin{array}{*{20}{c}} {TAR = \frac{{TP}}{{TP + FN}}} \end{array}$$

The FAR is the proportion that the system incorrectly accepts nonauthorized people, defined as

31 $$\begin{array}{*{20}{c}} {FAR = \frac{{FP}}{{FP + TN}}} \end{array}$$

By setting different threshold values for similarity scores (given by the face recognition systems), we obtain different TARs and FARs, resulting in a ROC curve. The AUC measures the 2D area underneath the ROC curve. TAR@FAR = X represents the TAR value when FAR equals X. Rank-1 is the probability that the similarity score of the same identity ranks first among all the identities.

Data were analyzed using SPSS (v.23.0, IBM Corp), R (v.4.1.1, R Project for Statistical Computing), C++ (v.11, Standard C++ Foundation) and Python (v.3.6, Python Software Foundation) with a designated significance level of 5%.

### Algorithm efficiency

Although considerable engineering effort is still needed to build a practical application, our main algorithm can run in real time. In detail, our algorithm takes approximately 7 ms, 14 ms and 4 ms per frame for face, eyelid and eyeball reconstruction, respectively, on one Intel i7 CPU and one NVIDIA 1080 GPU.

### Reporting summary

Further information on research design is available in the Nature Research Reporting Summary linked to this article.

## Online content

Any methods, additional references, Nature Research reporting summaries, source data, extended data, supplementary information, acknowledgements, peer review information; details of author contributions and competing interests; and statements of data and code availability are available at 10.1038/s41591-022-01966-1.

### Supplementary information

Supplementary Information Supplementary Note and Supplementary Table 1.

Reporting Summary

Supplementary Video A brief introduction to the DM.

Supplementary Data (1) The ophthalmologist’s diagnosis from the original videos, the ophthalmologist’s diagnosis from the DM-reconstructed videos and the normalized pixel error of the DM-reconstructed videos in relevant diagnosis comparisons of digital mask. (2) The questionnaire details of each patient in the empirical investigation of the DM.

## Figures and Tables

**Extended Data Table 1:**
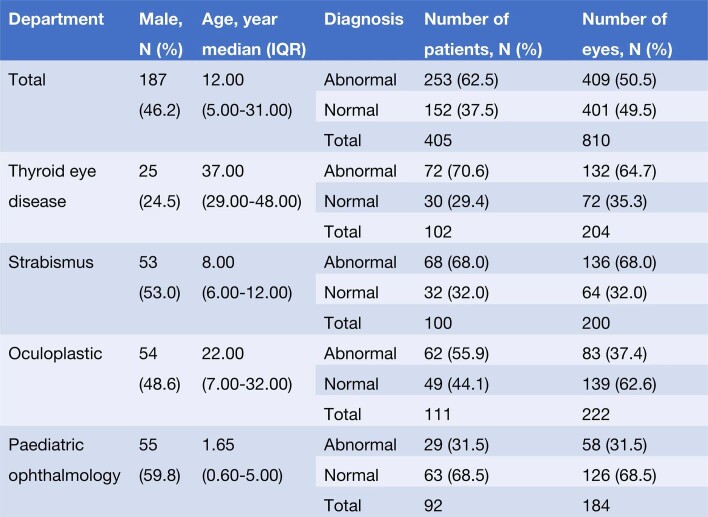
Characteristics of participants in the clinical trial

**Extended Data Table 2:**
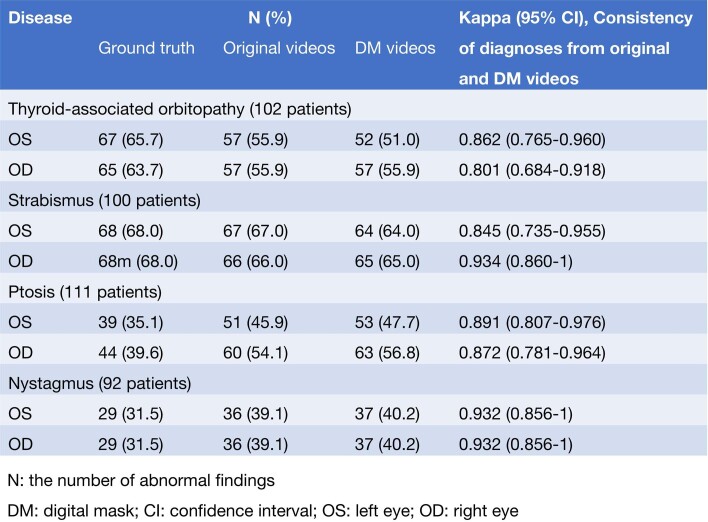
Primary outcomes of the diagnostic comparison in the clinical trial

**Extended Data Table 3:**
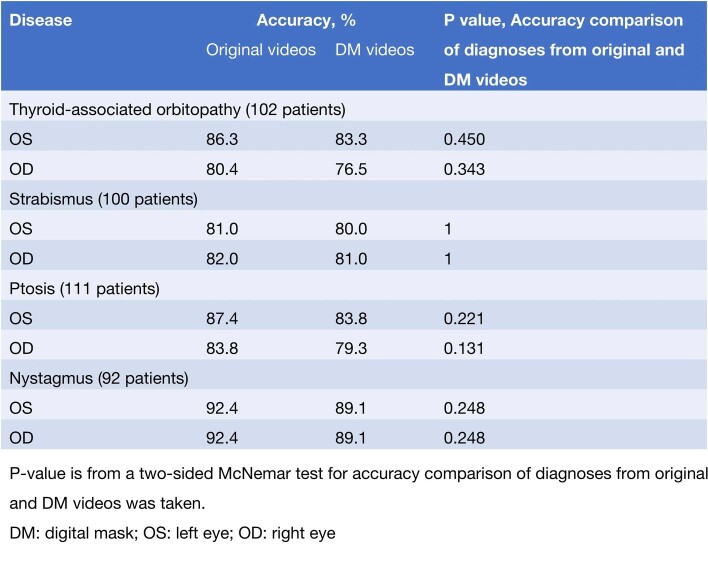
Secondary outcomes of the diagnostic comparison in the clinical trial

**Extended Data Table 4:**
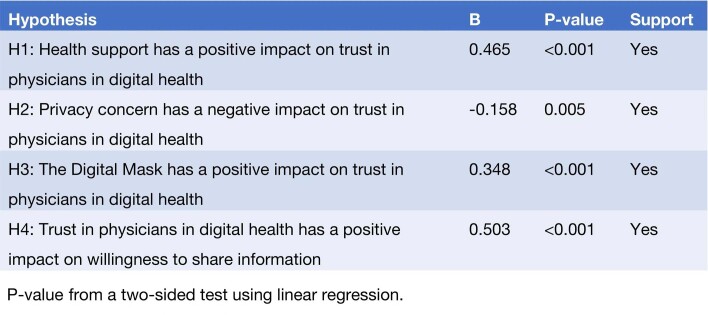
Prospective evaluation of the hypotheses included in questionnaire in the empirical investigation

**Extended Data Table 5:**
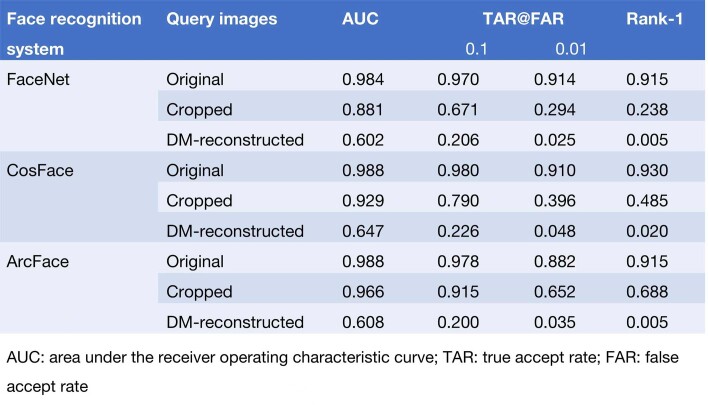
Performance of the face recognition systems in AI-powered reidentification validation

**Extended Data Table 6:**
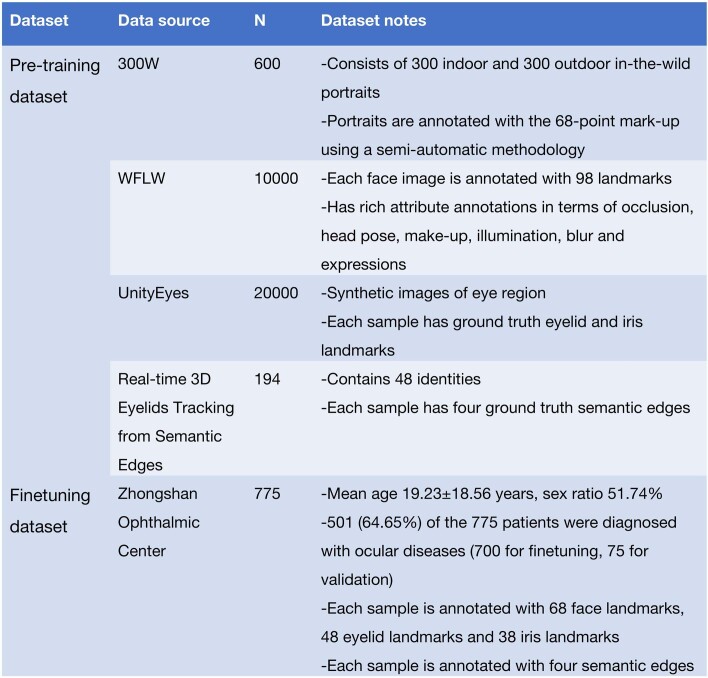
Characteristics of the training datasets

## Data Availability

The data that support the findings of this study are divided into two groups: shared data and restricted data. Shared data are available from the manuscript, references, supplementary data and video. Restricted data relating to individuals in this study are subject to a license that allows for use of the data only for analysis. Therefore, such data cannot be shared.

## Code availability

To promote academic exchanges, under the framework of data and privacy security, the code proposed by DM is available at https://github.com/StoryMY/Digital-Mask. In the case of non-commercial use, researchers can sign the license provided in the above link and contact H.L. or F.X. to access the code.
