# Supplementary material for: A digital mask to safeguard patient privacy
Source: Nat Med. 2022 Sep 15;28(9):1883–92. doi: 10.1038/s41591-022-01966-1 (PMC9499857; doi:10.1038/s41591-022-01966-1)
Supplement: Supplementary file 1 — Supplementary Note and Supplementary Table 1. [file 41591_2022_1966_MOESM1_ESM.pdf]

nature portfolio

https://doi.org/10.1038/s41591-022-01966-1

Supplementary information

A digital mask to safeguard patient privacy

In the format provided by the authors and unedited

Supplementary Information

Supplementary Note

The Digital Mask Guarantees Patient Privacy: a Prospective Study Protocol

Table of Contents

1. Background and Purpose……………………………………………………………..…..............2

2. Recruitment and Enrollment …………………………………………….……..…….............4

3. Evaluation Protocols and Research-related Risks..…………………………………….….............7

4. Statistical Analyses…………………………………………………………………….…............8

5. Certification of Personnel…………………………………………………………….……..........9

6. References…………………………………………………………………………….…............10

Chapter 1

Background and Purpose

1.1 Objectives

This study is a prospective trial with the following objectives:

- To assess the consistency of diagnoses based on DM videos and original videos.
- To evaluate the performance of diagnoses based on DM videos

1.2 Rationale of the Study

The study is conducted for the following reasons:

- The storage of facial images in patients' medical records poses privacy risks due to the sensitive nature of the personal biometric information that can be extracted from facial images.
- To minimize the risks of inappropriately disclosure of patient information, here we developed a new technology called the "Digital Mask" (DM), which is based on three-dimensional (3D) reconstruction and a deep learning algorithm to irreversibly erase identifiable features while retaining disease-relevant features needed for diagnosis.
- We will perform a prospective and observational trial to investigate whether the pathological signs retained by the digital mask are sufficient to disease diagnosis.

1.3 Summary of Study Protocol

Study flow chart

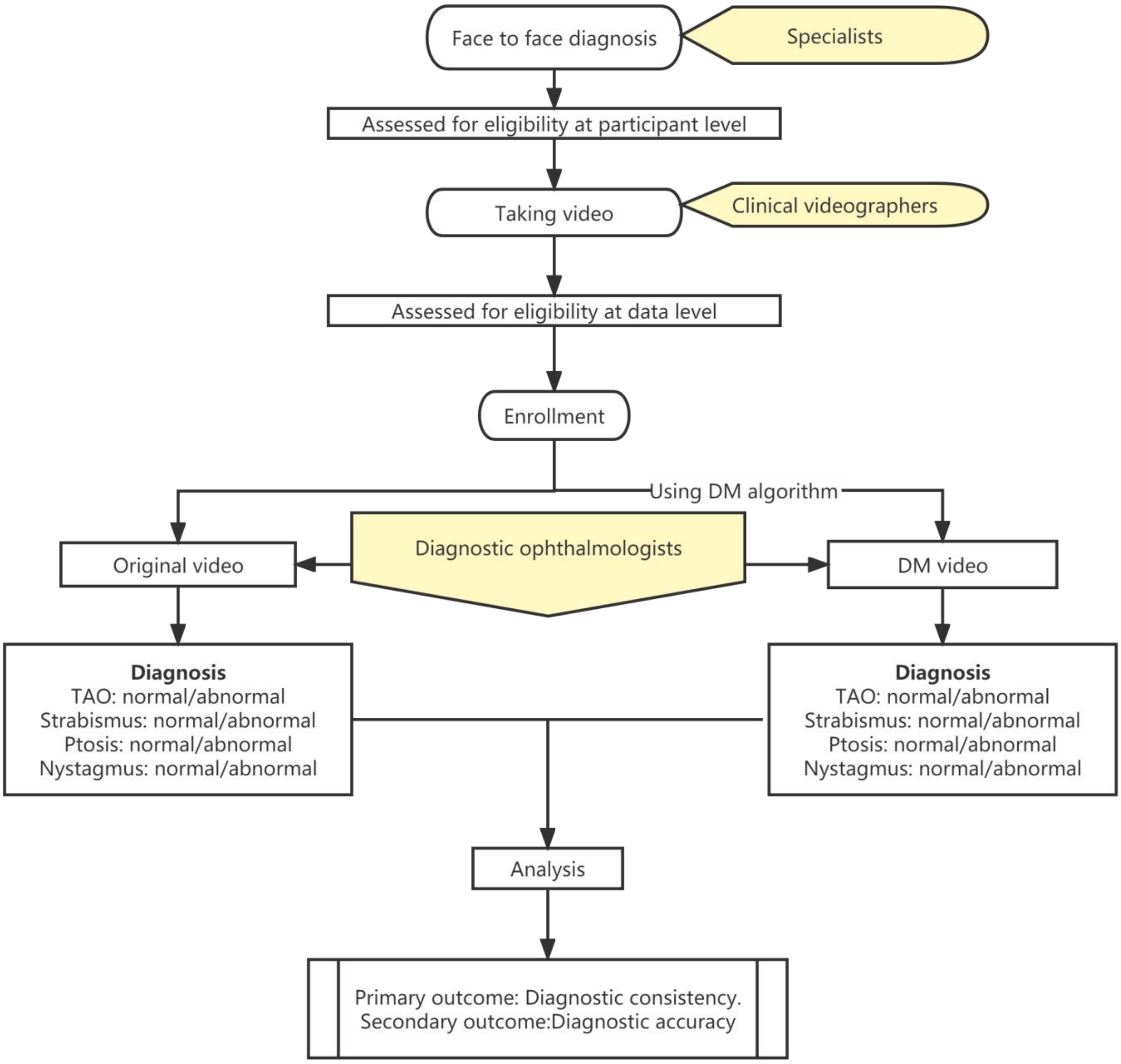

Study Sites: This study will be carried out in Zhongshan Ophthalmic Center of Sun Yat-sen University, China.

Major eligibility Criteria:

- One of the TAO, ptosis, strabismus, nystagmus, or normal will be diagnosed by a specialist.
- The quality of facial videos should be clinically acceptable.

Primary Outcome: The consistency of diagnoses from DM videos and original videos.

Secondary Outcomes: The accuracy of diagnoses from DM videos and original videos.

Chapter 2

Recruitment and Enrollment

2.1 Participants Recruitment

Participants will be recruited from Zhongshan Ophthalmic Center in China, and the outpatients from the following four departments are potentially eligible for the study: thyroid eye disease departments, oculoplastic departments, strabismus departments, and paediatric ophthalmology departments.

2.2 Informed Consent and Enrollment

This study will be discussed with the participants diagnosed with TAO, ptosis, strabismus, nystagmus, or normal by the specialist. Written informed consent will be obtained from the participant or at least one guardian. Once the participants are enrolled in the study, facial videos of appropriate ocular examinations will be taken.

2.3 Eligibility and Exclusion Criteria at Participant Level

2.3.1 Participates will be eligible for the study if all the following findings and conditions are met:

- One of TAO, ptosis, strabismus, nystagmus, or normal was diagnosed by a specialist;
- Informed consent signed by the participant or a parent or a legal guardian;
- Excluding unable to cooperate with eye movement examination or video taking (e.g. hyperactive or mentally retarded.).

2.3.2 **Diagnostic Criteria to Ground Truth**

A face-to-face diagnosis from a specialist is essential to provide the ground truth. The specialists from all four departments are required to have more than ten years of clinical work experience and diagnose according to international consensus diagnostic criteria. The specialists are allowed to obtain any clinical information related to the disease, including but not limited to conducting eye movement examinations, obtaining laboratory test data, asking about medical history, etc. A brief description is as follows:

①. **TAO**: TAO is diagnosed by positive responses of eyelid retraction and at least two of the following four sets of findings: Chemosis or eyelid edema; lid lag, or restrictive lagophthalmos[1,2].

②. **Ptosis:** Ptosis is defined as the upper eyelid falling to a position that is lower than normal (typically 1.0-2.0 mm below the superior corneoscleral limbus)[3]. The palpebral fissure distance is often evaluated by guiding the patient's eye fixation to a distant target[4]. The frontalis muscle, levator palpebrae muscle, and orbicular muscle are analyzed based on a series of movement guidelines to preliminarily explore the cause of ptosis; these movement guidelines include having the patient gaze upward and downward, maintain an upward gaze for 1 min and close his or her eyes tightly shut[5]. Additionally, the presence of brown ocular movements and jaw motion are all provided to aid in diagnosing ptosis[6].

③. **Strabismus**: Strabismus is characterized as the eyes not properly aligned with each other when looking at an object. The cover test and alternate cover test are used in diagnosing strabismus[7]. Because most people have exotropia but do not need treatment, we exclude exotropia when determining the diagnosis of strabismus. The test allows wearing glasses, especially in the case of patients with accommodative esotropia.

④. **Nystagmus**: Nystagmus is characterized as the eyes moving rapidly and uncontrollably; this movement can be observed and diagnosed during eye movement recording[8]. Additionally, compensatory head position and median zone are important features of nystagmus.

2.5 Examination and Shooting Procedures

2.5.1 Patient Information

The following patient information will be collected: sex, age, and ground truth.

2.5.2 Eye Examination of Each Disease

TAO: Nine-direction eye movement test.

Ptosis: Analysis of the frontalis muscle and levator palpebrae muscle.

Strabismus: Cover test and alternate cover test.

Nystagmus: Target gaze and tracking test.

2.5.3 Video Shooting Criteria

①. Different cameras can be used, including but not limited to Nikon 3500, Huawei p30, and Sony 4k

②. Video resolution is required to at least 2K.

③. It is recommended to shoot with a tripod.

④. These videos will be taken under room illuminance ranging from 300 to 500 l x.

⑤. The whole facial appearance of patients will be collected.

⑥. The patient's face in the first frame of video is required to be frontal and not be occluded by anything.

⑦. A sudden change of head pose should be avoided.

⑧. The patient's face is recommended to be located in the center of the screen.

⑨. The quality of facial videos should be clinically acceptable, videos will be excluded if they meet any of the following criteria: visible shaking, frame skipping, an incomplete shot of the patient's face, overexposure, and low light.

⑩. Initial quality control will be taken by the videographer, and followed by the other one or two staff for double-checking.

Chapter 3

Evaluation Protocols and Research-related Risks

3.1 Number

Scramble the number of DM videos, making it impossible to match the participants in the original videos with the ones in the DM videos.

3.2 Masking

- The diagnostic ophthalmologists will be blinded to participant ID;
- There is no overlap between specialists that performed face-to-face assessment, clinical videographers and ophthalmologists that performed video assessment;
- Ophthalmologists will make a diagnosis only based on the video (other clinical information like history or laboratory examination will not be presented);
- The specialists and ophthalmologists are blinded with respect to each other's diagnoses.

3.3 Diagnostic Protocols

- Each video will be independently diagnosed by three ophthalmologists from each of the four departments.
- A dichotomous diagnosis of abnormal (1) or normal (0) will be made for both the left eye and the right eye.
- Both the original video and DM video from the same participant will be diagnosed by the same ophthalmologist, under the blindness of participant ID.
- The diagnosis of DM videos will be made one week after the original video diagnosis.
- The time to complete the diagnosis of DM videos should be very close to that of original videos.

3.4 Research-related Harms

- There is no research-related harms.

Chapter 4

Statistical Analyses

4.1 Sample Size Estimate

For each eye, both the diagnosis from the original videos and the diagnosis from the DM-reconstructed videos are recorded and compared. If the two diagnoses are almost perfectly consistent, it suggests that the reconstruction would be precise enough in clinical practice.

Cohen's Kappa statistics are used to evaluate the agreement of diagnoses based on the original videos and the diagnoses based on the DM-reconstructed videos. Kappa is interpreted as recommended by Landis and Koch [9]: a kappa value of $\kappa < = 0.00$ is considered as poor, 0.00–0.20 = slight, 0.21–0.40 = fair, 0.41–0.60 =moderate, 0.61–0.80 = substantial and ≥ 0.81 almost perfect. The power was set at 0.9, the significant level was 0.025, and a one-sided test. Assuming k1=0.85, k0=0.6, the probabilities of abnormal findings were 0.3 to 0.7, then the sample size for each disease was at least 82 estimated using irr package in R 4.1.1 (The R Project for Statistical Computing, Vienna, Austria).

4.2 Data Locking

The confirmed dataset will be locked by the principal investigator and the investigators for statistical analysis. The verified data cannot be modified unless problems are raised after data locking, and investigators could modify data appropriately and record it after confirmation.

4.3 Statistical Analysis

Statistical analysis will be conducted with R.4.1.1 (The R Project for Statistical Computing, Vienna, Austria). In the clinical validation, characteristics of participants are described as frequency (proportion) for categorical variables; for continuous variables, means (standard deviation, SD) are reported if they are normally distributed, otherwise median (interquartile range, IQR). Cohen's Kappa statistics are used to evaluate the diagnostic consistency in the relevant diagnostic comparison. In addition, we measure the accuracies of diagnoses based on the original videos and diagnoses based on the DM videos compared to the ground truth and compare them using McNemar test.

Chapter 5

Certification of Personnel

5.1 Certification of Specialists that Performed Face to Face Assessment

The certification process for a specialist will include:

1. Having more than 10 years of clinical experience in the relevant ophthalmological subspecialty.
2. Employed as a senior professional title.

5.2 Certification of Clinical Videographers

The certification process for a clinical videographer will include:

1. Working as an ophthalmic technician for more than 3 years.
2. Proficient in using video shooting tools.
3. The certification examination will ensure that the staff is familiar with the study protocol.

5.3 Certification of Diagnostic Ophthalmologists

The certification process for ophthalmologists will include:

1. Having more than 5 years of clinical experience in the relevant ophthalmological subspecialty.
2. Licensed to practice medicine and surgery as subspecialists.

References

1. Bartley, G. B. & Gorman, C. A. Diagnostic criteria for Graves' ophthalmopathy. *American journal of ophthalmology* **119**, 792-795 (1995).

2. Gorman, C. A. *et al.* A Prospective, Randomized, Double-blind, Placebo-controlled Study of Orbital Radiotherapy for Graves' Ophthalmopathy. *Ophthalmology* **127**, S160-s171, doi:10.1016/j.ophtha.2020.01.031 (2020).

3. Latting, M. W., Huggins, A. B., Marx, D. P. & Giacometti, J. N. Clinical Evaluation of Blepharoptosis: Distinguishing Age-Related Ptosis from Masquerade Conditions. *Semin Plast Surg* **31**, 5-16, doi:10.1055/s-0037-1598188 (2017).

4. Meltzer, M. A. Management and surgery of congenital and acquired ptosis: edited by Donald J. Bergin, MD, San Francisco, American Academy of Ophthalmology, Continuing Ophthalmic Video Education, 1990, total running time: 39 minutes. Price: $65.00. *Survey of Ophthalmology* **36**, 318 (1992).

5. Díaz-Manera, J., Luna, S. & Roig, C. Ocular ptosis: differential diagnosis and treatment. *Curr Opin Neurol* **31**, 618-627, doi:10.1097/wco.0000000000000600 (2018).

6. Pearce, F. C., McNab, A. A. & Hardy, T. G. Marcus Gunn Jaw-Winking Syndrome: A Comprehensive Review and Report of Four Novel Cases. *Ophthalmic Plast Reconstr Surg* **33**, 325-328, doi:10.1097/iop.0000000000000780 (2017).

7. Wright, K. W., Spiegel, P. H. & Hengst, T. *Pediatric ophthalmology and strabismus*. (Springer Science & Business Media, 2013).

8. Abel, L. A. Infantile nystagmus: current concepts in diagnosis and management. *Clinical and Experimental Optometry* **89**, 57-65 (2006).

9. Landis JR, Koch GG. The measurement of observer agreement for categorical data. Biometrics. **33**, 159–74 (1977).

Supplementary Table 1. The MI-CLAIM checklist

Before paper submission		
**Study design (Part 1)**	**Completed: page number**	**Notes if not completed**
The clinical problem in which the model will be employed is clearly detailed in the paper.	■ See Introduction section (Page 5)	
The research question is clearly stated.	■ See Introduction section (Page 5)	
The characteristics of the cohorts (training and test sets) are detailed in the text.	■ See Methods section (Page 27)	
The cohorts (training and test sets) are shown to be representative of real-world clinical settings.	■ See Methods section (Page 27)	
The state-of-the-art solution used as a baseline for comparison has been identified and detailed.	☐	
**Data and optimization (Parts 2, 3)**	**Completed: page number**	**Notes if not completed**
The origin of the data is described and the original format is detailed in the paper.	■ See Methods section (Page 27)	
Transformations of the data before it is applied to the proposed model are described.	■ See Methods section (Page 27)	
The independence between training and test sets has been proven in the paper.	■ See Methods section (Page 27)	
Details on the models that were evaluated and the code developed to select the best model are provided.	■ We use validation dataset to select the best model (Page 27, Network Training section)	
Is the input data type structured or unstructured?	☐ Structured ■ Unstructured	
**Model performance (Part 4)**	**Completed: page number**	**Notes if not completed**
The primary metric selected to evaluate algorithm performance (e.g., AUC, F-score, etc.), including the justification for selection, has been clearly stated.	☐	Not applicable as our work do not purely focus on deep learning
The primary metric selected to evaluate the clinical utility of the model (e.g., PPV, NNT, etc.), including the justification for selection, has been clearly stated.	☐	Not applicable as our work do not purely focus on deep learning
The performance comparison between baseline and proposed model is presented with the appropriate statistical significance.	☐	Not applicable as our work do not purely focus on deep learning
**Model examination (Part 5)**	**Completed: page number**	**Notes if not completed**
Examination technique 1[a]	☐	Not applicable as our work do not purely focus on deep learning
Examination technique 2[a]	☐	Not applicable as our work do not purely focus on deep learning
A discussion of the relevance of the examination results with respect to model/algorithm performance is presented.	☐	Not applicable as our work do not purely focus on deep learning
A discussion of the feasibility and significance of model interpretability at the case level if examination methods are uninterpretable is presented.	☐	Not applicable as our work do not purely focus on deep learning
A discussion of the reliability and robustness of the model as the underlying data distribution shifts is included.	☐	Not applicable as our work do not purely focus on deep learning
**Reproducibility (Part 6): choose appropriate tier of transparency**		**Notes**
Tier 1: complete sharing of the code	■	
Tier 2: allow a third party to evaluate the code for accuracy/fairness; share the results of this evaluation	☐	
Tier 3: release of a virtual machine (binary) for running the code on new data without sharing its details	☐	
Tier 4: no sharing	☐	

PPV, positive predictive value; NNT, numbers needed to treat. [a]Common examination approaches based on study type: for studies involving exclusively structured data, coefficients and sensitivity analysis are often appropriate; for studies involving unstructured data in the domains of image analysis or natural language processing, saliency maps (or equivalents) and sensitivity analyses are often appropriate.
