# Supplementary material for: A digital mask to safeguard patient privacy
Source: Nat Med. 2022 Sep 15;28(9):1883–92. doi: 10.1038/s41591-022-01966-1 (PMC9499857; doi:10.1038/s41591-022-01966-1)
Supplement: Supplementary file 2 — Reporting Summary [file 41591_2022_1966_MOESM2_ESM.pdf]

nature portfolio

Corresponding author(s): HL, QD, and FX

Last updated by author(s): 2022.7.24

Reporting Summary

Nature Portfolio wishes to improve the reproducibility of the work that we publish. This form provides structure for consistency and transparency in reporting. For further information on Nature Portfolio policies, see our Editorial Policies and the Editorial Policy Checklist.

Statistics

For all statistical analyses, confirm that the following items are present in the figure legend, table legend, main text, or Methods section.

n/a	Confirmed	
☐	☒	The exact sample size (*n*) for each experimental group/condition, given as a discrete number and unit of measurement
☐	☒	A statement on whether measurements were taken from distinct samples or whether the same sample was measured repeatedly
☐	☒	The statistical test(s) used AND whether they are one- or two-sided<br>*Only common tests should be described solely by name; describe more complex techniques in the Methods section.*
☐	☒	A description of all covariates tested
☐	☒	A description of any assumptions or corrections, such as tests of normality and adjustment for multiple comparisons
☐	☒	A full description of the statistical parameters including central tendency (e.g. means) or other basic estimates (e.g. regression coefficient) AND variation (e.g. standard deviation) or associated estimates of uncertainty (e.g. confidence intervals)
☐	☒	For null hypothesis testing, the test statistic (e.g. *F*, *t*, *r*) with confidence intervals, effect sizes, degrees of freedom and *P* value noted<br>*Give P values as exact values whenever suitable.*
☐	☒	For Bayesian analysis, information on the choice of priors and Markov chain Monte Carlo settings
☐	☒	For hierarchical and complex designs, identification of the appropriate level for tests and full reporting of outcomes
☐	☒	Estimates of effect sizes (e.g. Cohen's *d*, Pearson's *r*), indicating how they were calculated

Our web collection on statistics for biologists contains articles on many of the points above.

Software and code

Policy information about availability of computer code

Data collection	No software was used during the data collection.
Data analysis	Data were analyzed using SPSS (version 23.0, IBM Corp, New York, USA), R (version 4.1.1, The R Project for Statistical Computing, Vienna, Austria), C++ (version 11, Standard C++ Foundation, Bellevue, Washington, USA), and Python (version 3.6, Python Software Foundation, Wilmington, Delaware, USA) with a designated significance level of 5%.

For manuscripts utilizing custom algorithms or software that are central to the research but not yet described in published literature, software must be made available to editors and reviewers. We strongly encourage code deposition in a community repository (e.g. GitHub). See the Nature Portfolio guidelines for submitting code & software for further information.

Data

Policy information about availability of data

All manuscripts must include a data availability statement. This statement should provide the following information, where applicable:

- Accession codes, unique identifiers, or web links for publicly available datasets
- A description of any restrictions on data availability
- For clinical datasets or third party data, please ensure that the statement adheres to our policy

The data that support the findings of this study are divided into two groups: shared data and restricted data. Shared data are available from the manuscript, references, supplementary data and video. Restricted data relating to individuals in this study are subject to a license that allows for use of the data only for analysis. Therefore, such data cannot be shared.

Field-specific reporting

Please select the one below that is the best fit for your research. If you are not sure, read the appropriate sections before making your selection.

☒ Life sciences ☐ Behavioural & social sciences ☐ Ecological, evolutionary & environmental sciences

For a reference copy of the document with all sections, see nature.com/documents/nr-reporting-summary-flat.pdf

Life sciences study design

All studies must disclose on these points even when the disclosure is negative.

Sample size	We prospectively evaluated the proposed technology in 405 patients with four different representative pathological ocular manifestations. In the sample size estimate of the clinical trial, the power was set at 0.9, the significance level was 0.025, and a one-sided test was used. Assuming k1=0.85 and k0=0.6, the probabilities of abnormal findings were 0.3 to 0.7, and the sample size for each disease was at least 82 estimated using the irr package in R 4.1.1 (The R Project for Statistical Computing, Vienna, Austria)
Data exclusions	Excluding unable to cooperate with eye movement examination or video taking (e.g. hyperactive or mentally retarded.).
Replication	The experiment was replicated in four pathological ocular manifestations. The sample size of each manifestation was sufficient (at least 92 people, 184 eyes). All attempts at replication were successful. In order to reproduce all experiments described in this paper, the code proposed by DM is available at https://github.com/StoryMY/Digital-Mask.
Randomization	There is one group in our study. Therefore, randomization is not applicable.
Blinding	The diagnostic ophthalmologists were blinded to participant ID; there was no overlap between specialists that performed face-to-face assessment, clinical videographers and ophthalmologists that performed video assessment; ophthalmologists made a diagnosis only based on the video (other clinical information like history or laboratory examination will not be presented); the specialists and ophthalmologists were blinded with respect to each other's diagnoses.

Reporting for specific materials, systems and methods

We require information from authors about some types of materials, experimental systems and methods used in many studies. Here, indicate whether each material, system or method listed is relevant to your study. If you are not sure if a list item applies to your research, read the appropriate section before selecting a response.

Materials & experimental systems

n/a	Involved in the study	
☒	☐	Antibodies
☒	☐	Eukaryotic cell lines
☒	☐	Palaeontology and archaeology
☒	☐	Animals and other organisms
☐	☒	Human research participants
☐	☒	Clinical data
☒	☐	Dual use research of concern

Methods

n/a	Involved in the study	
☒	☐	ChIP-seq
☒	☐	Flow cytometry
☒	☐	MRI-based neuroimaging

Human research participants

Policy information about studies involving human research participants

Population characteristics	The feasibility of the proposed technology was evaluated on a video dataset of patients in the clinical trial. From May 2020 to September 2021, 405 participants, 187 (46.2%) males, aged 4 months to 61 years who agreed to participate in the prospective study at the Digital Mask Program either by themselves or via their legal guidance. In total, 253 (62.47%) of the 420 patients were diagnosed with ocular diseases on the basis of face-to-face assessments of the patients' eyes.
Recruitment	Participants were prospectively recruited from Zhongshan Ophthalmic Center in China. Participates are eligible for the study if all the following findings and conditions are met:<br>1) One of thyroid-associated orbitopathy, ptosis, strabismus, nystagmus, or normal was diagnosed by a specialist;<br>2) Informed consent signed by the participant or at least one legal guardian;<br>3) Excluding unable to cooperate with eye movement examination or video taking (e.g. hyperactive or mentally retarded).
Ethics oversight	The research protocol and ethical review of this study was approved by the Institutional Review Board/Ethics Committee of the Zhongshan Ophthalmic Center. Consent was obtained from all individuals whose images are shown in figures or the video for publication of these images. Informed consent was obtained from at least one legal guardian of each infant, and the tenets of the Declaration of Helsinki were followed throughout this study.

Note that full information on the approval of the study protocol must also be provided in the manuscript.

Clinical data

Policy information about clinical studies

All manuscripts should comply with the ICMJE guidelines for publication of clinical research and a completed CONSORT checklist must be included with all submissions.

Clinical trial registration	NCT05058599
Study protocol	The study protocol can be accessed in the Supplementary information.
Data collection	The study was conducted in the outpatient clinic of thyroid eye disease departments, oculoplastic departments, strabismus departments, and pediatric ophthalmology departments. From May 2020 to September 2021, 405 outpatients were invited and 405 videos were collected.
Outcomes	Primary outcome of the relevant diagnostic comparison in the clinical trial is the consistency of diagnoses from DM videos and original videos. Cohen's Kappa statistics are used to evaluate the primary outcome. Kappa is interpreted as recommended by Landis and Koch 9: a kappa value of $\kappa <= 0.00$ is considered as poor, 0.00–0.20 = slight, 0.21–0.40 = fair, 0.41–0.60 =moderate, 0.61–0.80 = substantial and $\geq$ 0.81 almost perfect. The secondary outcome is the accuracy of diagnoses from DM videos and original videos. The accuracy is shown as the percentage of correctly diagnosed cases.
